# Parents of Child Psychiatric Patients Report More Adverse Childhood Experiences Compared with Community Samples

**DOI:** 10.3390/children11121427

**Published:** 2024-11-26

**Authors:** Adriana Altpeter, Andrea Dixius, Eva Möhler

**Affiliations:** 1 Department of Clinical Pharmacy, Saarland University, 66123 Saarbruecken, Germany; andrea.dixius@uni-saarland.de (A.D.); eva.moehler@uks.eu (E.M.); 2 SHG Clinic for Child and Adolescent Psychiatry, 66119 Saarbruecken, Germany; 3 Child and Adolescent Psychiatry, Saarland University Hospital, 66421 Homburg, Germany

**Keywords:** adverse childhood experiences, trauma, child, adolescent, trauma screen, PTSD

## Abstract

Adverse childhood experiences (ACEs) have already been associated, in some studies, with various diverse psychosocial abnormalities in later life. However, it is still unclear whether ACEs reported by biological parents differ from ACE scores in community samples. **Background/Objectives**: The aim of this study was to investigate the extent to which parents of a patient sample differ from a community sample in terms of reporting childhood experiences. In addition, the connection between parental negative traumatic experiences and their children’s reporting of these experiences should be examined in more detail. **Methods**: In total, 256 child psychiatric patients (73.8% female and 26.2% male) aged 4–18 years (mean [M] = 13.26 years, standard deviation [SD] = 2.73) were retrospectively examined for post-traumatic stress symptoms (using the CATS questionnaire). In addition, 391 caregivers, 316 of whom were biological parents, completed the ACE questionnaire on adverse childhood experiences. The frequencies of ACEs of the parents, the traumatic experiences of the patients and their cumulative occurrence were evaluated descriptively. **Results**: A total of 139 (73%) mothers reported at least one negative experience in childhood. In contrast, 65 fathers (52%) reported at least one negative experience in childhood. Mothers most frequently mentioned separation from a parent (38.7%), while fathers cited emotional abuse as the most frequent negative experience. These ACE scores were significantly higher than those reported from community samples. Post-traumatic stress disorder was diagnosed in 75 (29.3%) of the 256 patients. A total of 44.6% of children of mothers and 53.8% of children of fathers reporting at least one ACE showed a CATS score above the cut-off. **Conclusions**: Parents of child psychiatric patients show higher scores of adverse childhood experiences than a community sample with the same population background. Further empirical studies in parents of child psychiatric patients and a larger sample seem mandatory in the face of these results.

## 1. Introduction

Adverse experiences in early childhood are a critical factor in lifelong mental and physical health, both in adults and their children [1]. The Adverse Childhood Experience (ACE) questionnaire was developed as a composite self-report of abuse, neglect and domestic problems in childhood (before age 18). Abuse can occur in physical, sexual and psychological forms. Physical abuse is the intentional use of physical force against a child that results in or can result in physical injury [2]. Sexual abuse is any completed or attempted sexual act on a child [3]. Intentional behavior by a caregiver that conveys to a child that they are worthless, defective, unloved or unwanted is referred to as psychological abuse [4]. Neglect can have a medical, emotional and/or physical background and is based on the failure of a caregiver to meet the basic needs of a child [2].

These experiences can have long-lasting effects upon the subjected individuals. For example, high ACE scores are associated with long-term cardiovascular disease in later life or with negative metabolic changes in adulthood [5]. In addition to physical health, the psychological effects of ACEs are also of concern. These can be additive and combine with other developmental stressors (e.g., sexism, medical comorbidities, racism, etc.) to increase the risk of health conditions across the lifespan [6]. Likewise, ACEs can be passed down through generations, with a high number of parental ACEs having a cumulative negative effect [7]. Physical and psychological problems in the child can arise in the areas of temperament, academic performance, behavior and infant/toddler development [8].

The intergenerational transmission of adverse childhood experiences has also been described. The epigenetic transfer of ACEs may occur due to stress during pregnancy or mother–infant interactions [9,10]. Also, fathers with higher ACE scores reported more pregnancy-related anxiety than fathers with lower ACE scores at almost all time points during pregnancy measured in one study while still reporting depressive feelings during pregnancy [11]. Thus, ACEs can be passed on not only through interactions and parenting style but also through the role model function of parents. For example, children in families with multiple stressors are at the highest risk of high negative emotionality compared to families without stress [12].

Furthermore, it has been demonstrated that ACEs can have a negative effect on the health of adults [13]. The question of how these effects can influence the children of those affected will be investigated in the following paper. In previous studies investigating the relationship between traumatized parents and traumatized children, a clinical sample of refugees and their children showed a positive correlation between violence experienced by mothers in their childhood and violence experienced by children in childhood [14]. To extend these findings, we examine the question of which trauma-specific symptoms are reported by children whose parents experienced violence or other adverse childhood experiences.

## 2. Materials and Methods

Participants. The sample consisted of 256 patients and 391 caregivers/parents. In total, 316 parents completed the ACE questionnaire between January 2020 and July 2024. Within the child psychiatric patient sample, there were 189 female (73.8%) and 67 male (26.2%) participants. Patients from the following countries took part in this study: America (0.4%), Bulgaria (0.4%), Germany (94.9%), Malaysia (0.4%), Poland (0.4%), Romania (0.4%), Russia (0.4%), Syria (1.2%), Ukraine (1.2%) and Venezuela (0.4%). The average age at the time of completing the first questionnaire was 13 years (SD = 2.73).

Procedure. ICD-10 diagnoses were confirmed in a routine clinical procedure using standardized, disorder-specific diagnostic procedures. These diagnoses were assigned with the help of a collaborative medical and therapeutic assessment based on various appropriate diagnostic tools. The ICD-10 is the 10th revision of the WHO International Classification of Diseases and Related Health Problems. It is used by physicians and psychotherapists to code diagnoses and symptoms. The CATS questionnaires were completed by the patients themselves, while the ACE questionnaires were answered by caregivers or parents. Patients were asked to complete the following questionnaires in a quiet environment, accompanied by a therapist in case of extensive problems with the material or other issues, and caregivers could complete the questionnaires in the clinic or at home. Patients and caregivers could ask the trained therapists at any time if they had any questions or doubts. In addition, there was no time limit for completing the questionnaires, and patients and caregivers were informed about the nature, significance and scope of this study and the resulting requirements as part of the prior consent process. Only complete and assignable questionnaires were included in this data set.

Materials and Methods. Sociodemographic data such as the age, sex, mental diagnosis, country of origin and native language of the patients were considered in the survey and are described in Table 1.

ACE. The “Adverse Childhood Experiences Questionnaire” (ACE) is a valid, reliable and economical screen for the retrospective assessment of adverse childhood experiences and consists of 10 items on child maltreatment and problems in the parental home with yes or no answers [6,15]. A total score of 4 (maximum of 10) or higher is considered as having high-risk symptoms [16]. The German version was used and an overall score was calculated [15].

CATS and CATS-2. The “Child and Adolescent Trauma Screen” (CATS) is a short, freely accessible screening instrument directly based on the DSM-5 criteria for post-traumatic stress disorder (PTSD). It is a measure of potentially traumatic events and of post-traumatic stress symptoms [17,18]. In this study, the CATS Self-report (7–17 years) was used in most cases. If the patients were under 7 years old, the CATS Caregiver-report (3–6 years) was used. Post-traumatic stress symptoms (PTEs) are assessed using a 15-item structured PTE checklist. The PTE checklist follows the definitions of traumatic events in the DSM-5 and ICD-11 and includes items assessing natural disasters, serious accidents, experiencing or seeing violence at home or in the community, sexual abuse (off- and online), bullying, cyberbullying, traumatic loss, medical procedures and war. Patients who answered yes to at least one event are then assessed using 25 items that are rated on a 4-point Likert scale with the following anchor points: 0 = “Never”, 1 = “Sometimes”, 2 = “Often”, 3 = “Almost always”. The 25 items correspond directly to the diagnostic criteria for PTSD in the DSM-5 and PTSD and complex post-traumatic stress disorder in the ICD-11 [17]. The validation of the CATS showed good psychometric properties and has a proven good to excellent internal consistency of the symptom scales, with α ranging between 0.88 and 0.94 for the different versions [19].

Statistical analyses. The IBM SPSS Statistics program (IBM SPSS Statistics 27.0, 2021) was used for statistical analysis. A purely descriptive data analysis was carried out using the SPSS and Excel (Microsoft Excel, Version 16.82) programs. Regression analyses were not used. For this reason, it was not yet possible to use *p*-values and other parameters at the time of publication. Methodologically, only data from respondents who completed all questionnaires were used. Likewise, only the data of subjects who had both a CATS and at least one ACE, completed by a reference person, were used.

## 3. Results

Table 2 shows the distribution of diagnoses in the group of child psychiatric patients. Post-traumatic stress disorder was diagnosed in 75 (29.3%) of the 256 patients. The diagnosis of “moderate depressive episode” was attested 45 times (17.6%) and an adjustment disorder 40 times (15.6%). The fourth most common diagnosis was “other emotional disorder of childhood” (15; 5.9%). A further 31.6% was distributed among other diagnoses, which are described in detail in Table 2.

**Figure 1:**
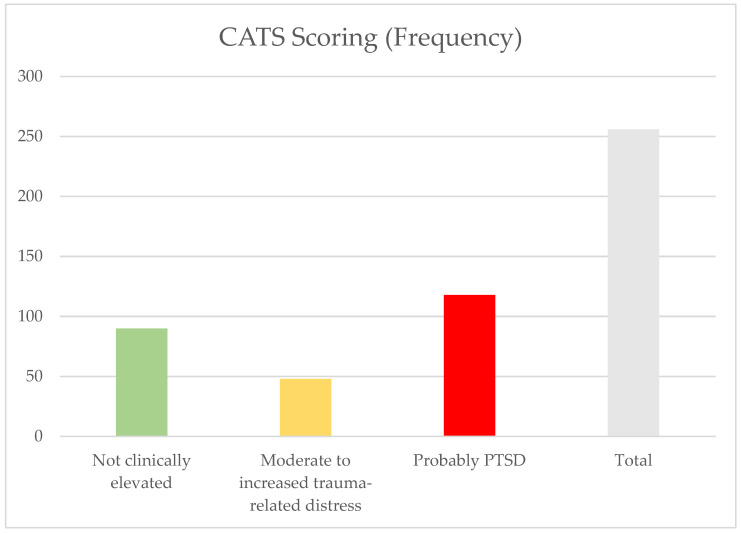
CATS scoring in child psychiatric patients.

Due to the length of this study, two different versions of the CATS questionnaires were used. For better clarity, codes 2 (moderate trauma-related symptoms) and 3 (increased stress symptoms) of the CATS were combined into “moderate to increased stress symptoms”. Of the 256 patients, 90 (35.2%) had a score >15 in the evaluation, which is not considered clinically noticeable. A total of 48 patients (18.8%) showed moderate to increased stress symptoms. Almost half of the patients (*n* = 118; 46.1%) reported high trauma-related stress symptoms, which, according to the authors, probably indicates PTSD. The frequency is shown in Figure 1.

**Figure 2:**
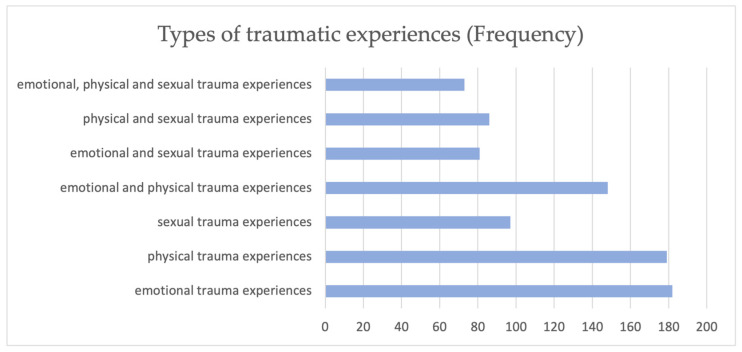
Types of traumatic experiences.

To classify the individual traumatic experiences, we divided them into “emotional trauma experiences”, “physical trauma experiences”, “sexual trauma experiences” and the various mixed forms. Items 6, 7, 12 and 14 (CATS) and 6, 7, 10, 11 and 14 (CATS 2) were combined into emotional trauma experiences. Physical trauma experiences included items 3, 4, 5 and 11 (CATS) and 3, 4 and 5 (CATS 2). Items 8 and 9 (CATS and CATS 2) represented sexual trauma experiences. The most frequently mentioned trauma experience was emotional abuse (*n* = 182). Patients least frequently reported all three types of traumas at the same time (*n* = 73). The classification of the types of traumas is shown in Figure 2.

ACE questionnaires were completed by the patients’ caregivers. In this study, only questionnaires of biological parents were considered (*n* = 316). The 75 questionnaires completed by the other named caregivers were not considered in more detail in this study. There were 191 questionnaires completed by mothers and 125 questionnaires completed by fathers. A total of 139 (72.8%) mothers reported at least one adverse childhood experience. In contrast, 65 fathers (52.0%) reported at least one ACE.

**Figure 3:**
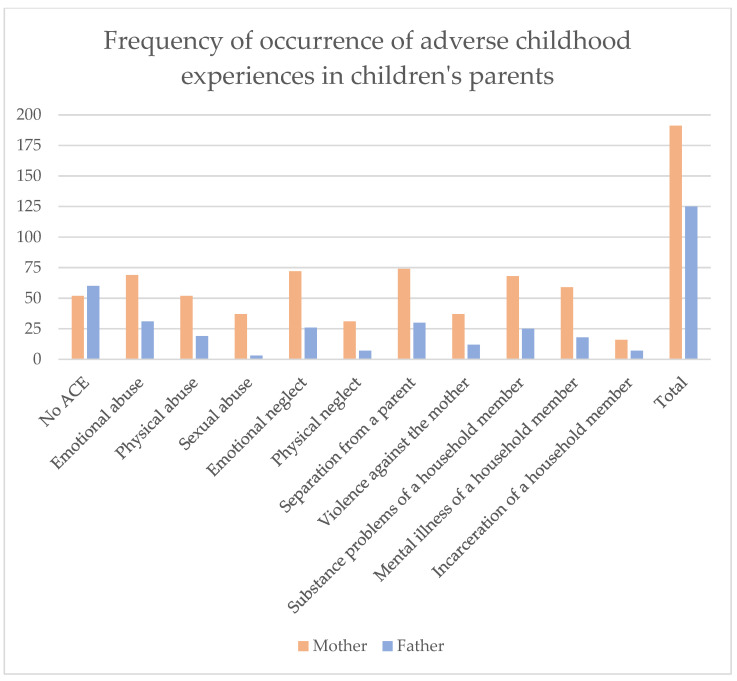
Frequency of occurrence of adverse childhood experiences in children’s parents.

Figure 3 and Table 3 show the prevalence of the individual negative childhood experiences. The most frequently mentioned ACEs among mothers were separation from a parent (38.7%), emotional neglect (37.7%), emotional abuse (36.1%) and substance problems of a household member (35.6%). The most common experiences reported by fathers were emotional abuse (24.8%), separation from a parent (24.0%), emotional neglect (20.8%) and substance problems of a household member (20.0%).

Table 4 provides an overview of the prevalence of combined adverse childhood experiences. Fifty-two mothers (27.2%) reported no such experiences, whereas more than two-thirds of the mothers reported at least one traumatic experience. Around half of the fathers (48.0%) reported no adverse childhood experiences.

A total of 33 mothers (17.3%) and 23 fathers (18.4%) reported one adverse childhood experience; 21 mothers (11.0%) and 12 fathers (9.6%) reported two adverse experiences, whereas 23 mothers (12.0%) and 11 fathers (8.8%) reported three adverse childhood experiences.

**Figure 4:**
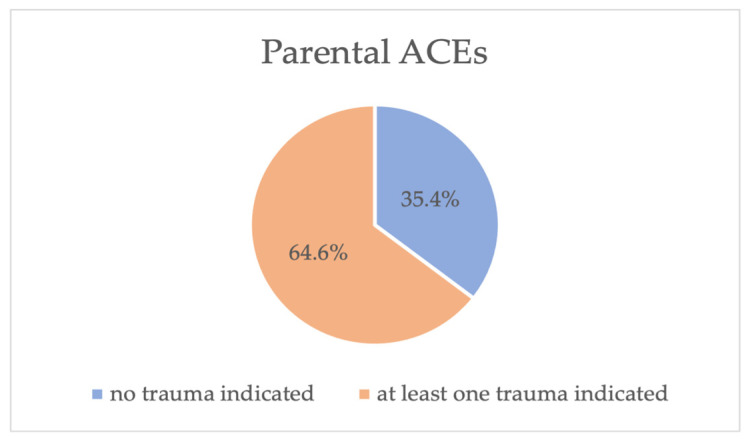
Parental ACEs.

A total of 62 mothers and 19 fathers belonged to the high-risk group of people who reported four or more adverse childhood experiences. A total of 139 mothers (72.8%) and 65 fathers (52.0%), i.e., 204 parents (64.6%), reported at least one adverse childhood experience. The distribution is described in Figure 4.

**Figure 5:**
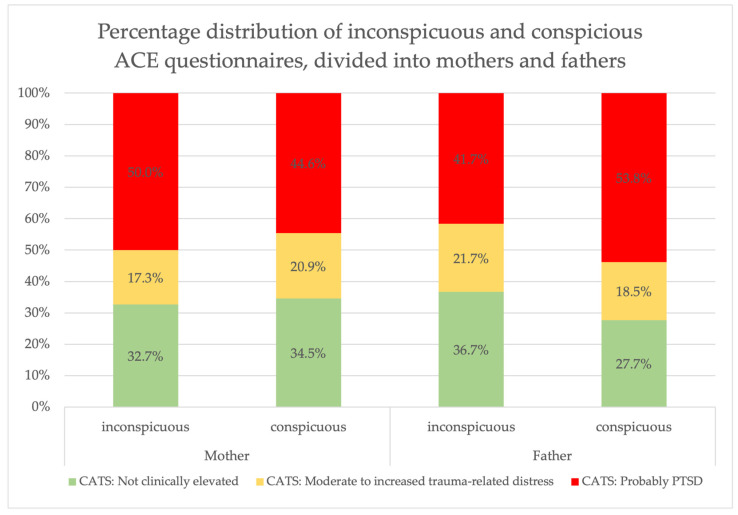
Percentage distribution of inconspicuous and conspicious ACE questionnaires, divides into monthers and fathers.

The connection between the patients’ CATS and the ACE forms of the biological parents is shown in detail in Figure 5. Among mothers who did not report any adverse childhood experiences, 50.0% of the patients reported probable PTSD, whereas 44.6% of patients whose mothers also reported at least one adverse childhood experience showed a CATS above the cut-off. For fathers, an inverse connection is indicated. A total of 53.8% of the patients reported probable PTSD if the father’s ACE questionnaire reported at least one item. In contrast, only 41.7% of the patients reported probable PTSD if the fathers did not report any adverse childhood experience.

## 4. Discussion

Previous studies report a supportive effect of ACE assessment on treatment planning and targeted interventions and an associated impact on psychological well-being and mortality risk [20]. It has also been shown that in the German population as a whole, 43.7% of subjects report at least one ACE [21]. In particular, those who reported four or more traumatic experiences in their childhood are more susceptible to later mental health impairments [22]. This corresponds to the results of other international studies [23].

In our study, the focus was not on the German population as a whole, but on a clinical child and adolescent psychiatric sample. In particular, the question of the connection between parental ACEs and childhood trauma was the focus of this study, showing that adverse childhood experiences occur more frequently in parents of children with psychiatric illnesses. A specifically strong relationship was found between a father’s ACEs and the child’s PTSD. This intergenerational transmission is in line with previous work reporting the transfer of a traumatic life event from parents to children [24,25,26], showing among other things increased impulsiveness and reduced emotional availability in parents with a history of abuse, which can negatively impact the parenting style. An altered perception of children’s signals and a change in neurobiological processing have also been reported in endocrinological and fMRI studies on mothers with a history of abuse [27,28,29]. Other studies have already shown that children in troubled families have an increased risk of negative emotionality [12]. In addition, negative experiences in childhood seem to have a significant influence on emotional processing in adult life [30].

Our findings are consistent with the studies mentioned. Furthermore, our results suggest that parents of child psychiatric patients report more adverse childhood experiences compared with community samples. In our survey, 64.6% of parents reported having experienced at least one ACE in their childhood. This is a considerably higher score than the one reported for the general population. When parents are divided into mother and father, the percentages also exceed the number of the total German population. An interesting result is the high ACE load of fathers. At least 44.6% of patients reported possible PTSD if the mothers also reported severe stress in childhood. For fathers, the percentage was slightly higher, at 53.8%. A possible explanation for this result could be that it is not only mothers that have an influence on the development of children, but fathers also determine this and act as role models for their children. It is possible that the different parental forms of ACEs also have different impacts and may promote transmission in different ways. Paternal transmission may occur via increased impulsiveness as described above [25]. While this phenomenon is likely to occur in both genders, impulsive fathers might be perceived and experienced by children as more threatening than impulsive mothers. Another gender difference was shown with regard to the type of reported experience: For example, sexual abuse is often reported by mothers, but not by fathers, while emotional neglect and emotional abuse are reported by both parents as two of the three most common forms. However, it should be noted that more mothers than fathers completed the questionnaires, so that the fathers’ reporting of ACEs, and therefore also the relation to child psychiatric symptoms, could change as the number of questionnaires increases.

The effects of individual ACE types also require further investigation, particularly in more diverse and representative samples. It is very important to consider the reported ACEs of parents and their children individually but in relation to each other. The experiences reported by parents could have different effects on the reports of children. Furthermore, examinations about the epigenetic heritability of traumatic experiences are required. Specifically, studies focusing on parent–child interaction, also taking neurobiological and endocrine responses into account, are required [31]. On another note, further demographic data, such as the living situation or other cultures with different language backgrounds should be included in future studies. In addition, longitudinal studies could provide information on the development of ACEs over time.

## 5. Limitations

One limitation of our study is the lack of a control group that examines the relationship between parental ACEs and childhood trauma in a non-clinical context. As both the ACE and the CATS are self-reported, there is a possibility that they were not filled out truthfully or that the items were not understood correctly. There is also a risk of questions not being answered honestly, possibly due to shame or lack of motivation. By using questionnaires, people with insufficient German language skills were systematically excluded, so people with German citizenship were significantly over-represented and people with a migration background are potentially under-represented. This ongoing study could help to shed more light on the connection between the traumatic experiences of parents and child psychiatric disease in their children.

## Figures and Tables

**Table 1:** Description of sample and sociodemographic characteristics.

Variable	*n* or M
Age (M, SD)	13.26 (2.73)
Sex (*n*, %)	
Male	67 (26.2)
Female	189 (73.8)
Country of Origin (*n*, %)	
America	1 (0.4)
Bulgaria	1 (0.4)
Germany	240 (94.9)
Malaysia	1 (0.4)
Poland	1 (0.4)
Romania	1 (0.4)
Russia	1 (0.4)
Syria	3 (1.2)
Ukraine	3 (1.2)
Venezuela	1 (0.4)
Native Language (*n*, %)	
Arabic	3 (1.2)
German	240 (93.8)
English	2 (0.8)
Italian	1 (0.4)
Polish	2 (0.8)
Portuguese	1 (0.4)
Romanian	1 (0.4)
Russian	2 (0.8)
Spanish	1 (0.4)
Ukrainian	2 (0.8)
Urdu	1 (0.4)

M = mean, n = number of patients, SD = standard deviation.

**Table 2:** Main diagnoses, International Statistical Classification of Diseases (ICD-10).

Diagnoses	ICD-10	Frequency (n)	Percent (%)
Mental and behavioral disorders due to use of alcohol, harmful use	F10.1	1	0.4
Acute polymorphic psychotic disorder with symptoms of schizophrenia	F23.1	1	0.4
Moderate depressive episode	F32.1	45	17.6
Severe depressive episode without psychotic symptoms	F32.2	1	0.4
Agoraphobia: with panic disorder	F40.01	2	0.8
Social phobias	F40.1	6	2.3
Specific (isolated) phobias	F40.2	2	0.8
Panic disorder	F41.0	1	0.4
Generalized anxiety disorder	F41.1	3	1.2
Mixed anxiety and depressive disorders	F41.2	5	2.0
Mixed obsessional thoughts and acts	F42.2	1	0.4
Acute stress reaction	F43.0	4	1.6
Post-traumatic stress disorder	F43.1	75	29.3
Adjustment disorders	F43.2	40	15.6
Other reactions to severe stress	F43.8	1	0.4
Mixed dissociative (conversion) disorders	F44.7	1	0.4
Anorexia nervosa	F50.0	5	2.0
Anorexia nervosa, active type	F50.01	2	0.8
Atypical anorexia nervosa	F50.1	1	0.4
Eating disorder, unspecified	F50.9	2	0.8
Emotionally unstable personality disorder	F60.31	2	0.8
Other habit and impulsive disorders	F63.8	1	0.4
Trans-sexualism	F64.0	1	0.4
Childhood autism	F84.0	1	0.4
Asperger syndrome	F84.5	1	0.4
Disorder of activity and attention	F90.0	6	2.3
Hyperkinetic conduct disorder	F90.1	8	3.1
Conduct disorder confined to the family context	F91.0	1	0.4
Unsocialized conduct disorder	F91.1	1	0.4
Socialized conduct disorder	F91.2	1	0.4
Oppositional defiant disorder	F91.3	4	1.6
Other mixed disorders of conduct and emotions	F92.8	2	0.8
Separation anxiety disorder of childhood	F93.0	1	0.4
Phobic anxiety disorder of childhood	F93.1	2	0.8
Other childhood emotional disorders	F93.8	15	5.9
Elective mutism	F94.0	1	0.4
Reactive attachment disorder of childhood	F94.1	1	0.4
Disinhibited attachment disorder of childhood	F94.2	2	0.8
Other childhood disorders of social functioning	F94.8	1	0.4
Other specified behavioral and emotional disorders with onset usually occurring in childhood and adolescence	F98.8	1	0.4
Attention deficit disorder without hyperactivity with onset usually occurring in childhood and adolescence	F98.80	2	0.8
Other specified behavioral and emotional disorders with onset usually occurring in childhood and adolescence	F98.88	1	0.4
Other problems related to lifestyle	Z72.8	1	0.4

**Table 3:** Prevalences of individual adverse childhood experiences (ACEs) of mothers and fathers and in total.

Diagnoses	Total Sample (*n*; %)	Mother		Father	
		Frequency (*n*)	Percent (%)	Frequency (*n*)	Percent (%)
ACE 1: emotional abuse	100 (31.6)	69	36.1	31	24.8
ACE 2: physical abuse	71 (22.5)	52	27.2	19	15.2
ACE 3: sexual abuse	40 (12.7)	37	19.4	3	2.4
ACE 4: emotional neglect	98 (31.0)	72	37.7	26	20.8
ACE 5: physical neglect	38 (12.0)	31	16.2	7	5.6
ACE 6: parental divorce/separation	104 (32.9)	74	38.7	30	24.0
ACE 7: witnessed domestic violence	49 (15.5)	37	19.4	12	9.6
ACE 8: alcohol and drug abuse in the household	93 (29.4)	68	35.6	25	20.0
ACE 9: mental illness in the household	77 (24.4)	59	30.9	18	14.4
ACE 10: incarcerated family member	23 (7.3)	16	8.4	7	5.6

**Table 4:** Prevalences of combined adverse childhood experiences (ACEs) of mothers and fathers and in total.

Number of ACEs	Total Sample (*n*; %)	Mother		Father	
		Frequency (*n*)	Percent (%)	Frequency (*n*)	Percent (%)
0	112 (35.4)	52	27.2	60	48.0
1	56 (17.7)	33	17.3	23	18.4
2	33 (10.4)	21	11.0	12	9.6
3	34 (10.8)	23	12.0	11	8.8
≥4	81 (25.6)	62	32.5	19	15.2

## Data Availability

The original contributions presented in this study are included in the article; further inquiries can be directed to the corresponding author.
